# Knowledge-transfer learning for prediction of matrix metalloprotease substrate-cleavage sites

**DOI:** 10.1038/s41598-017-06219-7

**Published:** 2017-07-18

**Authors:** Yanan Wang, Jiangning Song, Tatiana T. Marquez-Lago, André Leier, Chen Li, Trevor Lithgow, Geoffrey I. Webb, Hong-Bin Shen

**Affiliations:** 1 Institute of Image Processing and Pattern Recognition, Shanghai Jiao Tong University, and Key Laboratory of System Control and Information Processing, Ministry of Education of China, Shanghai, 200240 China; 2 Infection and Immunity Program, Biomedicine Discovery Institute and Department of Microbiology, Monash University, Melbourne, VIC 3800 Australia; 3 Monash Centre for Data Science, Faculty of Information Technology, Monash University, Melbourne, VIC 3800 Australia; 4 Infection and Immunity Program, Biomedicine Discovery Institute and Department of Biochemistry and Molecular Biology, Monash University, Melbourne, VIC 3800 Australia; 5 ARC Centre of Excellence for Advanced Molecular Imaging, Monash University, Melbourne, VIC 3800 Australia; 6 Informatics Institute, School of Medicine, University of Alabama at Birmingham, Birmingham, AL 35294 USA; 7 Department of Genetics, School of Medicine, University of Alabama at Birmingham, Birmingham, AL 35294 USA

## Abstract

Matrix Metalloproteases (MMPs) are an important family of proteases that play crucial roles in key cellular and disease processes. Therefore, MMPs constitute important targets for drug design, development and delivery. Advanced proteomic technologies have identified type-specific target substrates; however, the complete repertoire of MMP substrates remains uncharacterized. Indeed, computational prediction of substrate-cleavage sites associated with MMPs is a challenging problem. This holds especially true when considering MMPs with few experimentally verified cleavage sites, such as for MMP-2, -3, -7, and -8. To fill this gap, we propose a new knowledge-transfer computational framework which effectively utilizes the hidden shared knowledge from some MMP types to enhance predictions of other, distinct target substrate-cleavage sites. Our computational framework uses support vector machines combined with transfer machine learning and feature selection. To demonstrate the value of the model, we extracted a variety of substrate sequence-derived features and compared the performance of our method using both 5-fold cross-validation and independent tests. The results show that our transfer-learning-based method provides a robust performance, which is at least comparable to traditional feature-selection methods for prediction of MMP-2, -3, -7, -8, -9 and -12 substrate-cleavage sites on independent tests. The results also demonstrate that our proposed computational framework provides a useful alternative for the characterization of sequence-level determinants of MMP-substrate specificity.

## Introduction

Approximately 2% of the mammalian genome is devoted to encode proteases, i.e., proteolytic enzymes or peptidases^1, 2^. Proteases represent one of the largest enzyme families and play critical roles in cellular processes, such as cell development, apoptosis, immune response, and inflammation. In recent decades, biomedical research has advanced much of our understanding of the biological function of proteases, revealing the mechanisms associated with their digestion and breakdown of proteins into small fragments via the severing of peptide bonds^1–3^. Furthermore, a number of studies established that proteases can be used in a large number of biotechnological applications, including DNA extraction^4^, control of signaling pathways^5, 6^, and infection and manipulation of pathogens^7^. Given the diversity of their functional roles, proteases are also implicated in a number of human diseases. For example, the large family of serine proteases^8, 9^ includes specific regulators of inflammatory processes^10–13^ and plays a significant role in activating immune cells, including leukocytes, in inflammatory responses^12, 14^. Additionally, a number of proteases are linked to human cancers^6, 15, 16^ due to their ability to specifically target extracellular matrix proteins^6^ for degradation and some of their regulatory functions associated with tumor progression^16^.

Matrix metalloproteases (MMPs; also known as matrix metalloproteinases or matrix metallopeptidases) are zinc-dependent, calcium-containing hydrolases that belong to the metzincin group of metalloproteinases^17–21^. MMPs play a key role in proteolytic degradation of extracellular matrix proteins which are, in turn, generally involved in cellular communication and normal function. A characteristic sequence motif associated with these proteases includes the zinc-binding motif *HEXXHXXGXXH*, where *X* denotes any residues other than histidine (H), glutamic acid (E), or glycine (G)^18^. To date, 23 members of the MMP family have been characterized^20^, with previous studies establishing MMP involvement in a number of important biological processes, including cell proliferation^22^, migration^21, 23–25^, differentiation^26^, angiogenesis^26^, anti-inflammatory response^27–30^, vasoconstriction^31–34^, apoptosis^26^, and host defense^27^. MMP dysregulation is implicated in many diseases, including arthritis, ulcers, encephalomyelitis, and cancer. Given their important biological functions, MMP alterations, such as changes in expression levels or synthesis and degradation of the extracellular matrix, are likely to cause severe human health problems, including renal and cardiovascular diseases^35–38^. Moreover, given that an initial step in metastasis involves degradation of the cellular membrane, MMPs are implicated in poor prognoses associated with human cancers, with MMP-1, -2, -3, -7, -9, -13, and -14, all exhibiting elevated expression levels in primary tumors and/or metastases^22^. Experimental findings and even clinical trials a decade ago were very promising, but many of these studies were regarded negative or controversial and progress in the field slowed down^39, 40^.

More recently, high-throughput experimental techniques based on mass spectrometry, such as isotope-coded affinity tag^41^ and matrix-assisted laser desorption/ionization time-of-flight^42^, were successfully applied to identify MMP substrates^43–49^. The availability of these experimentally verified substrates has subsequently allowed tailored computational approaches for the prediction of potential MMP substrate-cleavage sites through development of tailored computational approaches^50–52^. However, while there are known substrate sequences and cleavage sites of certain types of MMPs, there is still limited substrate data available for others, including MMP-2, -3, -7, and -8. In this context, the discovery of novel substrate targets associated with these MMPs is expected to have significant impact, but remains a difficult task.

A variety of computational methods capable of predicting protease substrate-cleavage sites have been developed, including those of MMPs. PROSPER^50^ has been used to predict substrate-cleavage sites for 24 different proteases. It is one of the most powerful computational frameworks and is trained using support vector regression (SVR) based on a variety of sequence-based features. Additionally, PoPS^53^ and SitePrediction^54^ are two versatile statistical methods that allow users to predict potential substrate-cleavage sites of many proteases. Other methods are available as well, but tend to focus on prediction of substrate-cleavage sites for specific families or types of proteases. For example, several computational methods such as Cascleave (v1.0^51^ and v2.0^52^), Pripper^55^, CasPredictor^56^, GrasBCas^57^, CASVM^58^, and PCCS^59^, were developed to predict the cleavage sites of caspases and/or granzyme B. Consequently, the accurate prediction of diverse MMP substrate-cleavage sites remains an outstanding need and challenging problem, with limited studies focusing on methods for MMP substrate-cleavage site prediction^20^. Among the state-of-the-art methods, PROSPER is the only tool that can be used to predict MMP-2, -3, -7, and -9 substrate-cleavage sites. More recently, Kumar *et al*. developed CleavPredict^60^ based on position-weighted matrices to predict substrate-cleavage sites of 11 different MMPs. To facilitate this process, predictions by CleavPredict are integrated with other structural features, such as secondary structure and disordered regions. These contributions have been tempered by observations that the performance of these tools for predicting MMP substrate-cleavage sites was unsatisfactory according to experiments using independent test datasets: PROSPER only achieves an F-score of between 43% and 75% and a Matthews correlation coefficient (MCC) value of between 46% and 70% according to the original study^50^, while CleavPredict achieved an accuracy of between 39% and 72% and an area under the curve (AUC) value of between 0.76 and 0.89, depending on the type of MMP. These results highlight the need for new and effective means of improved prediction of MMP substrate-cleavage sites is highly desired.

In this context, it is worth noting that the amount of data of experimentally verified substrate-cleavage sites strongly depends on the associated MMP types. That is, there is very limited data for MMP-2, -3, -7, and -8 while there are much larger numbers of validated substrate-cleavage sites associated with MMP-9 and MMP-12. This motivates us to explore whether a knowledge transfer idea can be applied to ‘borrow’ the knowledge from the source domain (e.g. MMP-9 and MMP-12) to improve the prediction of cleavage sites of other MMPs (e.g. MMP-2, -3, -7, and -8) in the target domain. Our model presented herein thus adopts a transfer learning approach. The latter is a computational technique that can efficiently acquire knowledge from a domain *A* (the source domain; i.e. MMP-9 and MMP-12) and applies that knowledge to make predictions or inferences about domain *B* (the target domain; i.e. MMP-2, -3, -7, and -8), where *A* and *B* are two different but related domains^59^. Transfer learning has been successfully employed in many bioinformatics studies, such as biological-sequence analysis^61–66^, genetic data analysis^67^, system biology^68–71^, and biomedical applications^72–75^. Our results in this work show that although MMP-2, -3, -7, -8, -9 and -12 are different from each other, they share underlying common characteristics and the transferred cross-domain knowledge is indeed very useful and informative.

Additionally, we examined whether transfer learning could be further improved by combining it with feature-selection analysis, as amino acid sequence-based feature extraction and selection is related to the higher-dimensional learning problem. Not all initial features that are extracted end up being useful and relevant for the prediction. Accordingly, selection of most contributing features is often a pertinent consideration^76, 77^. However, the latter is not a straightforward task when it comes to training a transfer-learning model since two domains (source and target) will have to be dealt with. Accordingly, we performed cross-validation and independent tests to evaluate the performance of the transfer learning method and compared it a more traditional feature-selection method. Our results suggest that the transfer learning method can provide a robust performance, at least comparable to the latter method, indicating that the cross-domain knowledge transfer is a promising method for dealing with substrate-cleavage site prediction of MMPs with limited substrate data. In summary, our proposed transfer-learning-based method is a useful and complementary approach to existing studies of protease substrate cleavage site prediction. Our method proves most useful in cases where cleavage site data is limited. All source codes of our proposed method, as well as the benchmark datasets used in this study, are freely available and part of the supplementary material at http://lightning.med.monash.edu/tl/.

## Results

### Analysis of sequence-level determinants of MMP-substrate specificity

**Figure 1:**
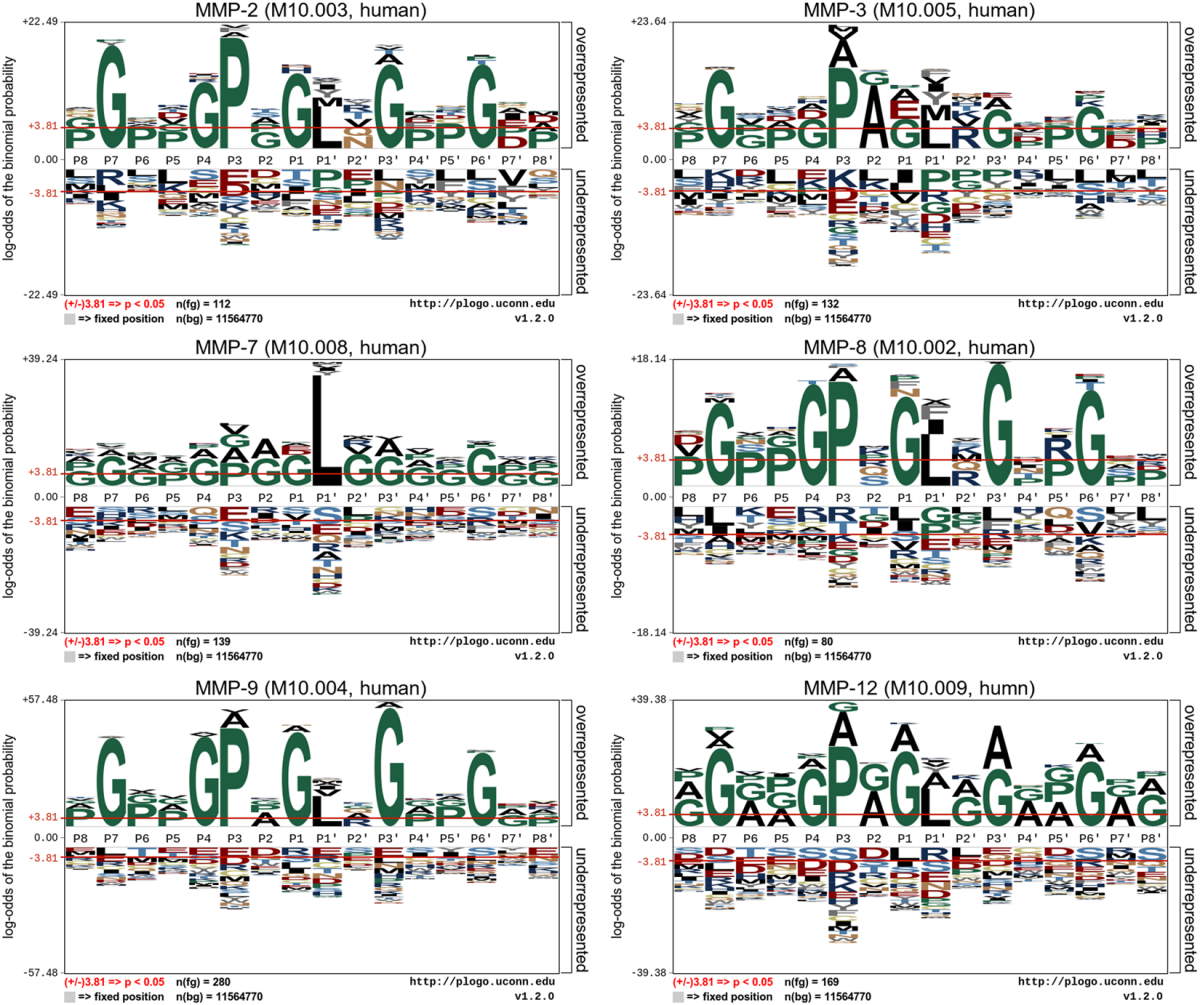
Sequence-logo representations of the occurrences of amino acid residues surrounding the substrate-cleavage sites (from P8 to P8′ positions) of MMP-2, -3, -7, -8, -9, and -12. Sequence logos were generated using the pLogo program^78^. The red horizontal lines on the pLogo plots denote the statistical significance threshold (*p* = 0.05).

Our first goal was to better understand the sequence-level determinants associated with MMP substrate-cleavage sites and to explore the efficiency of transfer-learning techniques in this line of research. By using curated MMP-substrate datasets, we analyzed the occurrences of amino acid residues surrounding the P8-P8′ sites. To identify common patterns among different MMP substrate-cleavage sites from both source and target domains, we subsequently rendered sequence-logo representations using the pLogo program^78^. As shown in Fig. 1, even though the residue distribution clearly varied among different MMP substrate-cleavage sites, they still exhibited similar sequence patterns. Remarkably, glycine was significantly overrepresented at the P7, P4, P1, P3′, and P6′ positions, proline was overrepresented at the P3 and P5′ positions, and leucine was overrepresented at the P1′ position (*p* < 0.05; Fig. 1), which we note is consistent with previous findings^79^.

We also observed significant overrepresentation of acidic residues surrounding the substrate-cleavage sites of multiple MMPs, including those at positions P7, P4, P3, P1, P3′, and P6′. While a predictive sequence motif was not readily apparent, we found that MMP substrate-cleavage sites were preferentially located in regions characterized by depletion of arginine residues in the N-terminal region and lysine and histidine in both the N- and C-terminal regions, with enrichment of acidic residues to a lesser extent (Fig. 1). Notably, while other machine-learning algorithms require additional training data, the substrate-cleavage site patterns shared by the six MMPs enabled us to use the transfer-learning framework to train the cleavage site prediction models. We would like to note that the heterogeneity of sequence patterns presented in Fig. 1 is not uncommon; in fact, heterogeneity has been reported in other studies in the literature. Here, the sequence logo plots were generated using the state-of-the-art sequence log software program pLogo^78^. This program contrasts itself with other traditional logo software in that it essentially relies upon residue frequencies to graphically scale character heights, by generating probabilistic sequence logos whose characteristics are scaled relative to their statistical significance.

### The overall framework

**Figure 2:**
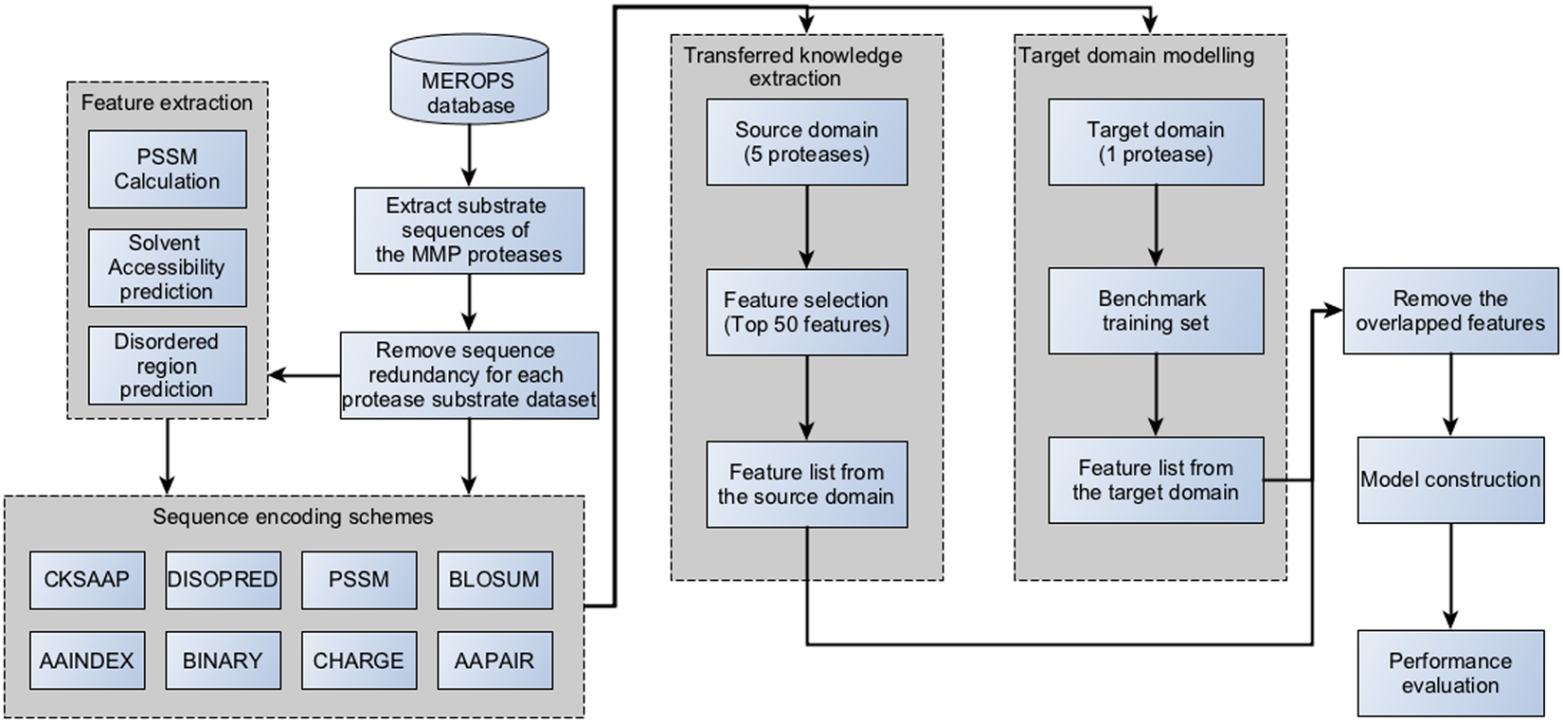
Workflow of the proposed transfer-learning framework.

In the experiments, each of the six datasets (namely MMP-2, -3, -7, -8, -9 and -12) was used as the test data in the target domain. To build the model based on knowledge-transfer learning, the data of the other five MMPs were used as the knowledge data in the source domain. An illustration of the proposed transfer-learning framework for the substrate-cleavage site predictions of MMP-2, -3, -7, -8, -9 and -12 is provided in Fig. 2. There are four major stages in the development of this framework: data preprocessing, feature encoding, model construction, and performance evaluation. During the data preprocessing stage, the CD-HIT^80^ algorithm was used to cluster homologous sequences with ≥70% sequence identity in the datasets and to reduce sequence redundancy, which can potentially lead to biased model training. Positive and negative samples were extracted with a ratio of 1:3 from the substrate datasets of MMP-2, -3, -7, -8, -9 and -12 using a sliding window of 16 amino acids (P8-P8′). Subsequently, eight feature-encoding schemes were used to generate the input feature sets used for model training. Models were then constructed for MMP-2, -3, -7, -8, -9 and -12, respectively, using the proposed transfer-learning method to predict their substrate-cleavage sites. In order to benchmark and compare the performance of the transfer learning method, we built a baseline model that was built after merging the substrate data of all six MMPs. This method served as our “baseline” and did not discriminate the knowledge extracted from the source domain or the target domain; in other words, the baseline method did not benefit from a knowledge-transfer procedure. Detailed descriptions of feature encoding, feature selection, transfer learning-based model construction and parameterization, and performance evaluation can be found in the “Materials and methods” section.

### Predictive performance of transfer learning-based models for substrate-cleavage site prediction of MMPs from the source domain

As aforementioned, the effective application of transfer learning requires high-fidelity transfer of the knowledge. In our case, this implies that the extracted common knowledge of MMPs from the source domain must be sufficient for the prediction of substrate-cleavage sites of MMPs in the target domain. To examine this, the predictive performances of SVR models of MMP-2, -3, -7, -8, -9, and -12, which were trained using the extracted common knowledge of MMPs in the source domain, were examined for their ability to predict substrate-cleavage sites of respective MMPs using five-fold cross-validation tests.

Eight different encoding schemes were used to generate a variety of features that describe the knowledge of substrate cleavage sites of the MMPs (See the “Sequence-encoding schemes” section for details). These sequence encoding schemes have proved useful for the prediction of protease substrate cleavage sites and other post-translational modification sites^50–52, 81^. Overall, a total of 4461 initial features (See Table 1 for a full list) were extracted for encoding the positive and negative cleavage sites of the six MMPs (please refer to the Materials and Methods section for details of positive and negative sites). As some of the extracted initial features are noisy and irrelevant for the prediction, we subsequently applied the minimum redundancy maximum relevance (mRMR) algorithm^82^ to select the top ranked features. In this work, 50 top ranked features were selected to encode substrate cleavage sites of MMPs from the source domain. Such features represent the extracted knowledge of substrate-cleavage sites of MMPs from the source domain, which are to be transferred to other MMPs in the target domain. For all the eight feature-encoding schemes (including Binary, PSSM, AAindex, BLOSUM, CKSAAP, DISO, CHR, and AAC) (Table 1), after the mRMR feature selection, the majority of features were selected from the Binary-encoding scheme, followed by the PSSM scheme. Both sequence encoding schemes have been found to be particularly useful for the prediction of protease cleavage sites in previous studies^50–52^. Our results here confirm that they indeed provide an enriched set of selected features, highlighting the usefulness of Binary features and evolutionary information in the form of PSSM for the prediction of substrate cleavage sites^50–52^ and also other types of protein post-translational modification sites^76, 77, 83^.

**Table 1:** A list of features extracted for each feature-encoding scheme.

Feature-encoding scheme	Feature dimensionality	Description
AAindex	1024	Physicochemical properties retrieved from the AAIndex database for each residue of the segment.
AAPair	20	The amino acid composition of the segments surrounding cleavage sites.
Binary	336	Binary encoding scheme extracting the position-specific information for each residue of the segments.
BLOSUM	336	The sequence information reflecting the similarity between two segments.
CHARGE-hyd	9	Charge and hydrophobicity information for amino acids around the cleavage site.
CKSAAP	2400	Composition of k-space residue pairs in the segment.
DISOPRED	16	Native disordered region of each segment.
PSSM	320	Evolutionary information of each segment.

**Figure 3:**
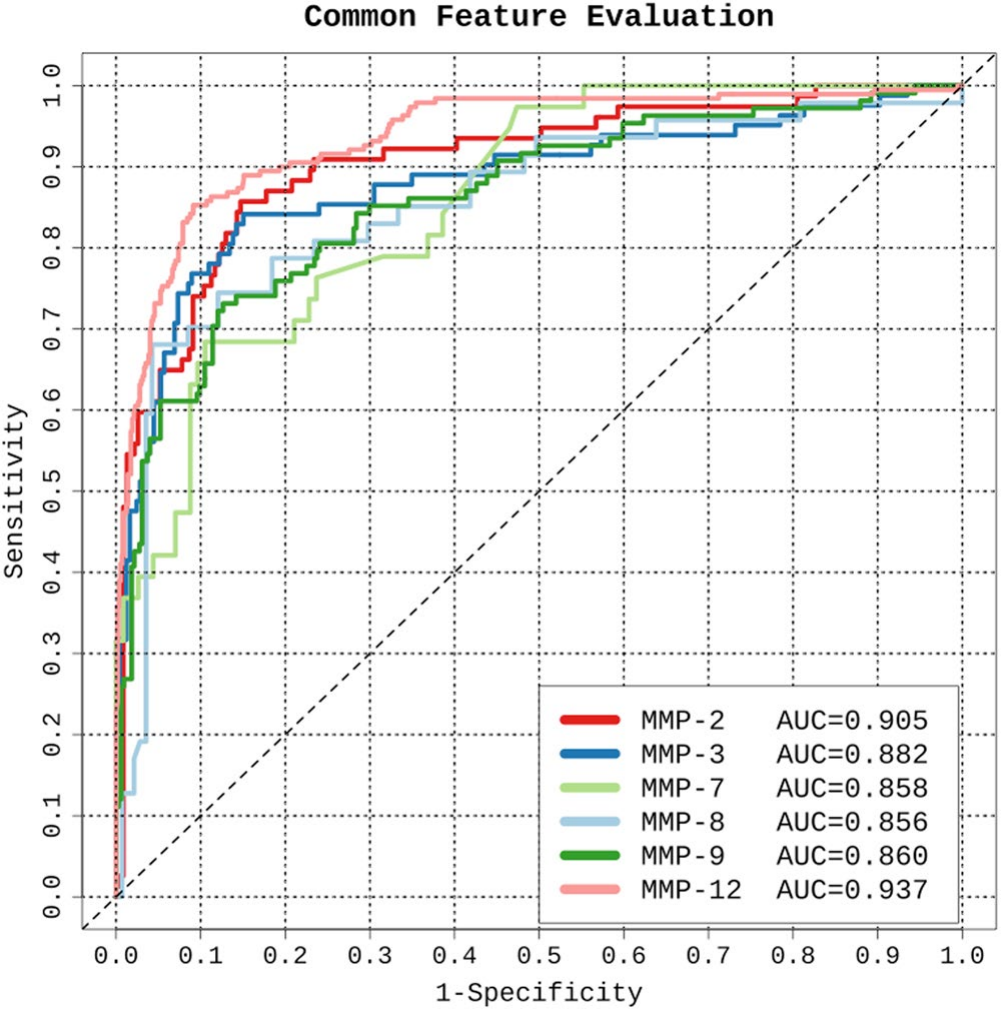
Performance of SVR models trained using the extracted common knowledge of MMPs in the source domain for the substrate cleavage site prediction of a corresponding MMP in the target domain.

To demonstrate the predictive performance of SVM models trained using the extracted common knowledge and evaluate their fidelity in retaining such knowledge, receiver operating characteristic (ROC) were derived and AUC values calculated (Fig. 3). In particular, SVR models trained using the extracted common knowledge achieved AUC values ranging from 0.856 to 0.937 for substrate-cleavage site prediction of the six MMPs. These results suggest that the top 50 features extracted from the source domain may well capture regularities that hold across multiple MMPs and hence such common features can be useful for the cleavage site prediction of other MMPs following knowledge transfer learning.

### Performance comparison of transfer-learning and baseline methods

In this section, we discuss and compare the performances of the transfer-learning and the baseline methods for the prediction of substrate cleavage sites of MMP-2, -3, -7, -8, -9, and -12, based on five-fold cross-validation tests.

For the transfer-learning method, the final features used for training the predictive models of each MMP included both common knowledge extracted from the source domain and other novel features extracted from the target domain. The latter features were selected using the mRMR algorithm^82^. The number of extracted features for each sequence encoding scheme varied for the six MMPs. While a significantly lower number of features from the target domain were extracted and chosen for MMP-7, four sequence encoding schemes have relatively larger numbers of selected features: Binary, CKSAAP, PSSM, and BLOSUM. On the other hand, the baseline method used the top 100 features selected by the mRMR algorithm to build the prediction model.

**Figure 4:**
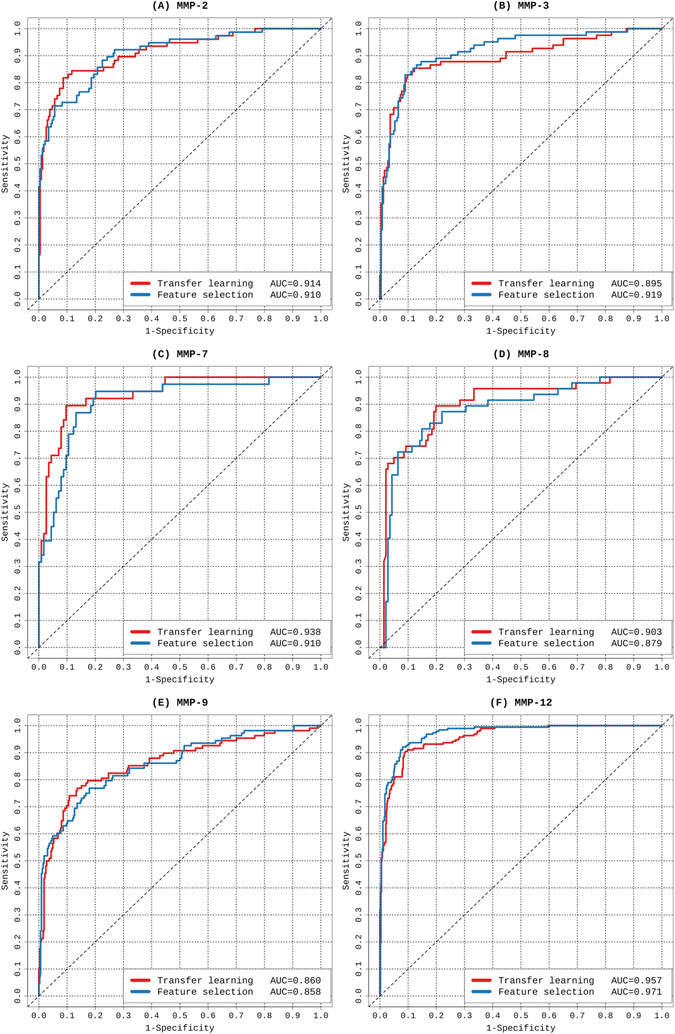
ROC curves of transfer learning and feature selection-based models for the prediction of substrate-cleavage sites for MMP-2, -3, -7, -8, -9, and -12. The results were evaluated based on five-fold cross-validation tests.

**Figure 5:**
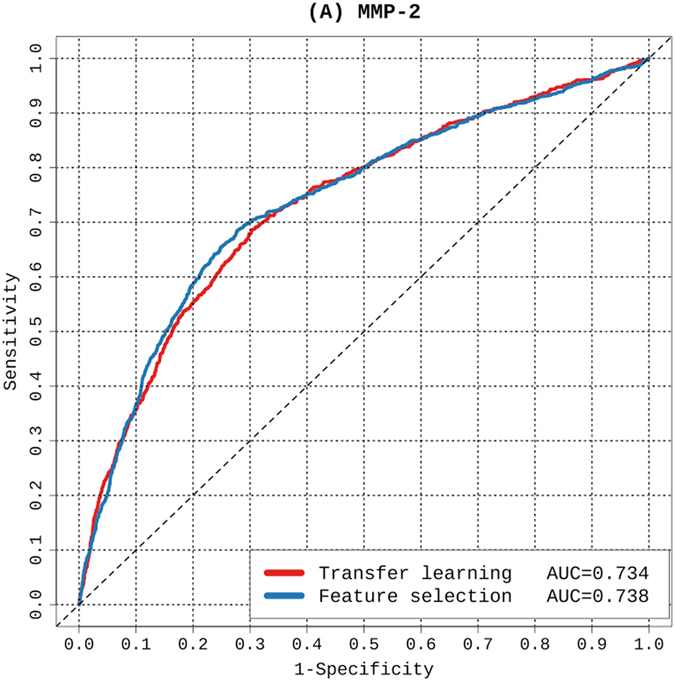
ROC curves of transfer learning and feature selection-based models for the prediction of substrate-cleavage sites for MMP-2. The results were evaluated based on independent test.

The predictive performances of the transfer-learning and baseline methods were evaluated based on five-fold cross-validation tests for all six MMPs (Fig. 4 and Table 2). As can be seen, the transfer-learning method achieved slightly lower AUC scores for the MMP-3 and MMP-12 substrate-cleavage site predictions than the baseline method. In contrast, for substrate-cleavage site predictions of MMP-2, -7, -8, and -9, the transfer-learning method achieved an outstanding performance with AUC values 0.914, 0.938, 0.903, and 0.860, respectively. As a comparison, the baseline method achieved AUC values 0.910, 0.910, 0.879, and 0.858, respectively. In terms of accuracy, the transfer learning method achieved a tangible performance improvement, i.e. it achieved an increase in Accuracy of nearly 5% for MMP-7 and 3% for MMP-2, respectively. In terms of sensitivity, the performance improvement is clearer. For example, the transfer learning method achieved an increase in sensitivity of 10.4%, 11.4% and 6.5% for MMP-2, 7 and 9, respectively (Table 2). Altogether, these results suggest that through effective integration of the common knowledge shared by multiple proteases, extracted from the source domain, the transfer-learning method indeed shows great promise for providing a superior or, at the very least, competitive predictive performance when compared to that of the baseline method. Lastly, we performed an independent test, and the prediction performances of the proposed transfer learning methods and baseline method are shown in Fig. 5. We note the transfer learning method achieved an AUC value of 0.734, which is competitive and comparable to that of the baseline method (AUC = 0.738).

**Table 2:** Predictive performance of the transfer-learning method and baseline method evaluated based on the five-fold cross validation tests.

MMP type	Model*	Accuracy (%)	Sensitivity (%)	Specificity (%)	F-score (%)	MCC
MMP-2	TL	87.987	83.117	89.610	77.576	0.697
	BL	85.390	72.727	89.610	71.338	0.616
MMP-3	TL	88.110	82.927	89.738	77.714	0.699
	BL	88.110	82.927	89.837	77.714	0.699
MMP-7	TL	89.474	89.474	89.474	80.952	0.744
	BL	84.868	71.053	89.474	70.130	0.600
MMP-8	TL	85.638	74.468	89.362	72.165	0.626
	BL	85.106	72.340	89.362	70.833	0.609
MMP-9	TL	84.954	70.370	89.815	70.046	0.600
	BL	83.333	63.889	89.815	65.714	0.548
MMP-12	TL	90.263	91.053	90.000	82.381	0.764
	BL	90.658	92.632	90.000	83.215	0.776

*Model: TL, Transfer-learning method; BL, Baseline method.

**Figure 6:**
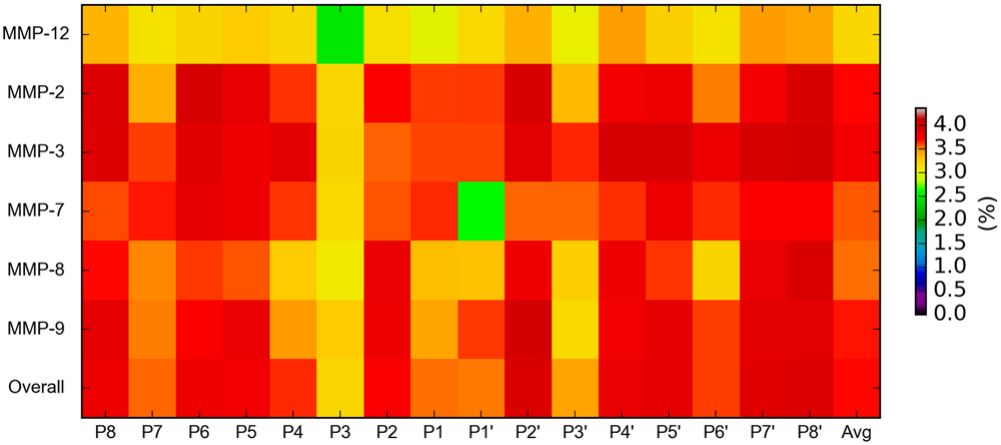
Heatmaps of cleavage entropies of MMP-2, -3, -7, -8, -9, -12, and the overall MMPs. The cleavage entropies of P8-P8′ positions surrounding the cleavage sites were calculated. The average values over all the P8-P8′ positions for each MMP were also calculated (indicated as “Avg”). Red colors indicate residue positions with larger cleavage entropy values (i.e. the corresponding protease has a higher cleavage specificity), while light yellow or green colors indicate residue positions with smaller cleavage entropy values (i.e. the corresponding protease has a lower cleavage specificity).

In order to better understand why the transfer learning method performed worse than the baseline method when predicting MMP-3 and MMP-12 substrate cleavage sites, we calculated the MMPs’ cleavage entropies^84^, a quantitative measure of their specificities. Our calculations were based on the sequences of cleaved substrates (P8-P8′ positions) obtained from the MEROPS database. The cleavage entropy results of all six MMPs are shown as horizontal heatmaps in Fig. 6 (from the top line to the bottom line we show cleavage entropies of MMP-12 (MEROPS ID M10.009), MMP-2 (M10.003), MMP-3 (M10.005), MMP-7 (M10.008), MMP-8 (M10.002), and MMP-9 (M10.004); from left to right: residue positions P8 to P8′). As can be seen, MMP-12 (MEROPS ID M10.009, the top line) has consistently lower cleavage entropy values compared to other MMPs, reflecting that this MMP cleaves target substrates with less specificity. Thus, it is understandable that the common knowledge obtained from other MMPs in the source domain is not readily transferrable to predict MMP-12 substrate cleavage sites. In contrast, MMP-3 (MEROPS ID M10.005, the third line from the top) displays (on average) the largest cleavage entropy values over the range of positions and consequently more stringent specificity as compared to all other MMPs. It is therefore conceivable that the knowledge transferred from other proteases to aid the learning of MMP-3 substrate cleavage sites effectively reduces the predictive performance. Taking all of these results together, it seems that transferred knowledge is useful in most cases but ineffective for predicting cleavage sites of MMPs at both extreme ends of the spectrum (high vs. non-specific).

## Discussion

In this work, we present a new transfer-learning framework for the prediction of MMP substrate cleavage sites and validate its usefulness by applying this framework to learn the knowledge from the source domain (MMP-9 and MMP-12) to improve the prediction of cleavage sites of other MMPs (MMP-2, -3, -7, and -8) in the target domain. Benchmarking experiments indicate that such framework is robust and particularly attractive when predicting cleavage sites of MMPs with limited training data. Overall, our results indicate that this new framework provides a useful alternative for the characterization of sequence-level determinants of MMP-substrate specificity.

When using machine-learning techniques to extract knowledge of value from datasets, a typical assumption (and also a prerequisite) is that there should be a sufficient amount of well-annotated training data to enable the construction of a robust and reliable prediction model. However, in many bioinformatics applications related to biological data mining, this assumption often fails due to intrinsic limitations of the underlying experimental techniques and the amount of the generated experimental data that is generated through such techniques. In this study, we focused on providing efficient solutions for the accurate prediction of substrate-cleavage sites of MMP-2, -3, -7, -8, -9, and -12, for which a limited number of experimentally validated cleavage sites have been previously reported. We developed two models, which were based on a transfer learning method and a baseline method in combination with conventional feature selection, respectively, and compared their predictive performances for each of the six MMPs using benchmark datasets.

Here, we have provided a useful, complementary and alternative approach to predict substrate-cleavage sites of MMPs, based on knowledge transfer learning. Our work adds value to and complements existing approaches, in particular when dealing with insufficient data and/or datasets with limited sizes, where other methods recurrently fail. In the future, we expect sufficiently large heterogeneous cleavage site data to become available through distinct experimental approaches (e.g. cleavage site data, high-throughput proteomic approaches *vs*. low-throughput gel-based assays at the full-length substrate sequence level for each individual MMP) and it will be worth investigating their potential impact on the prediction performance of cleavage sites.

For added clarity, we would also like to emphasize several major differences between this work and our previous PROSPER work^50^:

(i) The datasets used are different: The current work used substrate cleavage datasets of the MMP proteases; while PROSPER represents a generic approach that can be used to predict potential cleavage sites of 23 protease types;
(ii) Extracted features and selection techniques are different: The current work extracted several other types of features that are different from those features used by PROSPER, including AAindex features, BLOSUM features, AAPair features, CHARGE-hyd features, and CKSAAP features (Table 1). In addition, the current work utilised the mRMR feature selection technique, which is different from the mean decrease Gini index (MDGI) of the random forest (RF) algorithm used by PROSPER;
(iii) Integrations and model training strategies of the extracted features are different: The current work was developed based on the knowledge transfer learning strategy by transferring the knowledge learned from the source domain to the target domain; while the previous PROSPER work was based on the supervised learning of extracted features to train the models;
(iv) The research purposes are different: The current work is aimed at providing a useful alternative approach based on transferring useful knowledge learned from limited datasets, which complements with existing studies; while the primary aim of the PROPSER work was to provide a publicly available bioinformatics tool for computational prediction of multiple proteases.

Altogether, although both PROSPER and our transfer learning-based approach used support vector regression algorithms as the primary resource to train the prediction models for protease substrate cleavage sites, there exist major differences between PROPSER approach and our new transfer learning-based approach, presented in this work.

Our results presented herein demonstrated a MMP-specific predictive performance. Moreover, for MMP-3, -7, -8, and -9 substrate-cleavage site prediction, the transfer learning method outperformed the baseline method, highlighting the contribution of common knowledge extracted from the MMPs in the source domain to the cleavage site prediction of each individual MMPs. We anticipate this proposed transfer-learning-based framework will greatly facilitate the prediction of substrate-cleavage sites and further our understanding of the substrate specificity of MMPs. More generally, it provides a useful and complementary strategy to approach tasks associated with biological predictions using a limited supply of training samples. Lastly, while we recommend the proposed models be refined when more experimentally validated substrate-cleavage sites become available, this study provides a valuable method for the accurate prediction of substrate-cleavage sites. We expect that our findings and the proposed strategies will be inspirational and valuable for a number of biotechnology and biomedical applications where extraction of domain-common and specific knowledge is often required.

## Materials and Methods

### Non-redundant datasets

In this study, all experimentally verified substrates and their substrate-cleavage site annotations were extracted from the MEROPS database, which is a comprehensive, integrated knowledgebase for proteases, substrates, and inhibitors^85^. To avoid potential over-fitting, we performed sequence-homology reduction in the extracted substrate datasets using the CD-HIT^80^ program with a 70% sequence-identity threshold. To ensure proper machine-learning-based model training and performance assessment, we only considered MMPs that had at least 50 experimentally validated substrate-cleavage sites at the time of this study. The above filters resulted in a final set of six MMPs, 210 substrate sequences, and 942 cleavage sites. When the substrate dataset of a MMP was used as the dataset in the target domain, the remaining five MMP datasets were used as the dataset in the source domain. Table 3 provides a statistical summary of the MMP-specific substrate datasets used in this study.

**Table 3:** Statistical summary of MMP-specific substrate datasets used in this study.

Protease name	MEROPS ID	Number of substrates	Number of known cleavage sites
MMP-8; Matrix Metalloproteinase 8	M10.002	23	85
MMP-2; Matrix Metalloproteinase 2	M10.003	35	115
MMP-3; Matrix Metalloproteinase 3	M10.005	44	132
MMP-7; Matrix Metalloproteinase 7	M10.008	42	142
MMP-9; Matrix Metalloproteinase 9	M10.004	43	290
MMP-12; Matrix Metalloproteinase 12	M10.009	23	178

To extract the positive (i.e. cleavage sites) and negative (i.e. non-cleavage sites) peptide sequences, we further truncated the substrate sequences using a local sliding window, 16 residues in length, where the cleavage site was symmetrically flanked by eight upstream and eight downstream residues. Previous studies have shown the presence of important residue positions that might be involved in the substrate recognition of MMPs, for example, P4 or P3 to P2′ or P3′ positions for substrate recognition and P5-P4′ positions from protein structure point of view for substrate binding^50, 86^. In the current work, we employed a uniform window size of 16 amino acids (i.e. P8-P8′) to include extended neighbouring sequence environments that potentially could have an influence on the substrate determination. The number of negative samples was much larger than that of positive samples, which could lead to biased model training in favor of negative samples. To address this data imbalance, we adopted a re-sampling strategy using a ratio of 1:3 between the positive and negative samples as previously suggested^50^.

Additionally, non-cleavage sites needed to be accurately predicted as being solvent inaccessible, given that residues located in the core of the protein structure would likely be inaccessible to proteases^87^. Therefore, to facilitate the selection of reliably negative samples, we used the NetsurfP^88^ software, which allowed us to predict solvent accessibility of the P1 residues in substrate proteins. The solvent-inaccessible (shown as ‘b’ in the output of NetsurfP) P1 residues that were annotated as cleavage sites would more frequently be selected as reliable negative samples.

To evaluate the performance of the proposed transfer learning approach, we further constructed a dataset for independent testing. We first attempted to split our data into a training and a testing dataset. However, the resulting testing dataset was too small to provide any meaningful independent test results. To address this issue for MMP-2 substrates, we constructed an independent test dataset using the latest version of MEROPS (Release 11.0) that only recently had been largely extended by experimental substrate cleavage data for MMP-2. After removing the overlapping and homologous sequences (by clustering sequences using CD-HIT at the 70% sequence identity) from the training dataset, we obtained 714 sequences with 1,433 cleavage sites as the independent test set for MMP-2.

### Sequence-encoding schemes

Sequence-encoding schemes play an important role in determining the predictive performance of machine-learning-based models. Here, we used eight different sequence-encoding schemes for training SVR models based on a combination of various types of features. The sequence-encoding schemes and their corresponding feature dimensions are shown in Table 1. The window size of the sample, *L* (or the length of the segment) in this work was 16, and the total dimension for AAindex, AAPair, Binary, BLOSUM, CHARGE-Hyd, CKSAAP, DISOPRED, and PSSM was 4461, meaning that for each sample there is a 4461-dimensional input for the SVR. Detailed descriptions of these encoding schemes can be found in previous work^50, 81^. Here, we applied a computational tool to convert the amino acid segments to numerical vectors, including over ten different kinds of encoding schemes, as proposed by Chen *et al*.^81^. The detailed information and dimensionality of each encoding scheme is described below.

#### AAindex

AAIndex^89^ (v9.1) is a database containing 544 indices of amino acid physicochemical properties, such as alpha-CH chemical shifts and hydrophobicity index. Previous studies demonstrated that 64 of 544 indices are informative and beneficial for predictive tasks in a number of computational bioinformatics studies^90^. Therefore, we chose these 64 high-quality indices for use in our study. As a result, the AAindex-derived features were encoded as a *L* × 64 = 16 × 64 = 1024-dimensional real-valued vector, where *L* is the length of the segment.

#### AAPair

AAPair, also called Amino Acid Composition (AAC), has been widely used in a variety of protein-sequence analyses and predictions, including substrate-cleavage site prediction^50^ and ubiquitination-site prediction^76, 81^. In this study, it was used to calculate the frequencies of amino acids surrounding the cleavage site. Therefore, each segment in our datasets was encoded as a 20-dimensional vector.

#### Binary representation of amino acids in the segments

The amino acids flanking cleavage and non-cleavage sites were accounted for by using the binary sequence-encoding scheme as previously described^50–52^. Each amino acid residue was transformed into a 20-dimensional binary vector, alphabetically-sorted, and was represented by a combination of ones and zeros, e.g., alanine (10000000000000000000), cysteine (01000000000000000000), etc. Apart from the 20 standard amino acids, we used ‘00000000000000000000’ to represent unnatural amino acids when necessary. Each segment in our datasets containing *L* = 16 amino acids was encoded as a *L* × 21 = 16 × 21 = 336-dimensional binary vector.

#### BLOSUM62 matrix

The BLOSUM62 matrix was used to extract primary sequence information. A vector of *L* × 21 elements was used to represent each segment in our datasets, where *L* is the length of the segment and 21 represents the 20 standard amino acids and an additional one representing non-conserved amino acid residues. Therefore, the BLOSUM-derived features for a segment of length *L* = 16 comprised a 16 × 21 = 336 dimensional vector^76^.

#### CHARGE-Hyd

We also used CHARGE-Hyd^76, 91, 92^ to calculate the charge and hydrophobicity of each amino acid segment contained in our datasets. Extracted information for each 16-residue segment included the mean net charge, the aromatic content, and the charge:hydrophobicity ratio, with each feature consisting of a three-dimensional vector. The resulting dimensionality of the CHARGE-Hyd-based features for each segment in our dataset was 3 × 3 = 9^76^.

#### CKSAAP

Composition of k-space Amino Acid Pair (CKSAAP)^81^ encoding was used to calculate the amino acid pairwise frequencies for segments contained in our datasets. When *k* = 0, this indicates that there are 400 amino acid pairs (i.e., AA, AC, AD, …, YY) and that the encoded vector can be defined as:

1 $${(\frac{{N}_{AA}}{{N}_{Total}},\frac{{N}_{AC}}{{N}_{Total}},\frac{{N}_{AD}}{{N}_{Total}},\ldots ,\frac{{N}_{YY}}{{N}_{Total}})}_{{\rm{400}}}$$

The values of *N*_*Total*_ for a 16 amino acid fragment were 15, 14, 13, 12, 11, and 10 for spaces *k* = 0, 1, 2, 3, 4, and 5, respectively. When a fragment is located at the N- or C-terminus, the value of *N*_*Total*_ was adjusted accordingly. In our study, the CKSAAP-encoding scheme was used with *k* = 0, 1, 2, 3, 4, and 5. Accordingly, the dimensionality of the resulting feature vector was 2400^81, 93^.

#### DISOPRED

Previous studies indicated that incorporation of natively disordered regions^50–52^ can be useful for protease substrate-cleavage site prediction. Therefore, we accounted for native-disorder features using DISOPRED^94^ to predict the native profiles of substrate sequences. DISOPRED outputs the predicted probability for each residue being disordered (denoted by ‘*’) or ordered (denoted by ‘.’). Native-disorder features were encoded as a *L* × 1 = 16 × 1 = 16-dimensional vector based on the probabilities associated with the corresponding residues.

#### Position-specific scoring matrix (PSSM)

PSSM represents the occurrence probability for each type of amino acid at each corresponding position. PSSM profiles are widely used in many biological data analyses as a primary sequence-derived feature. In our study, PSSM profiles were generated for each sequence in the dataset using PSI-BLAST^95^ against the UniRef90 protein database to yield an essential sequence-derived input feature.

### Feature selection

Given that the initial features extracted from multiple sources are heterogeneous, we performed feature selection to remove any noisy and/or misleading features by using the mRMR^96^ algorithm, which ranks the importance of all features. mRMR is able to rank features based on their relevance according to the response variables (labels) and redundancy among the features. Therefore, optimal candidate features can be identified and selected after performing mRMR calculations, thereby enhancing predictive performance.

### Model training and parameterization

Support vector machine (SVM) is a supervised machine learning technique that has been widely applied to solve a variety of classification problems. In practice, SVM has two modes: the classification mode and the regression mode^97^. In this study, we used the regression mode, i.e. SVR, to train models for the prediction of MMP substrate-cleavage sites. In particular, for SVR, the real-valued prediction output value associated with each sample (either positive or negative) can be readily transformed to a classification outcome by applying a prediction cutoff value. The probability score generated by the SVR model can serve as a useful confidence metric for each predicted sample. Due to its attractive advantage, SVR has been used by several protease cleavage site prediction studies^50–52^ and was chosen as the baseline algorithm for our transfer learning-based approach, as the choice of the baseline algorithms for transfer learning is not the focus or goal of this study. Here, we used the LibSVM^97^ package with regression mode to output a quantitative score for each residue from the substrate sequences. The SVR classifiers were trained using the rational basis function kernel. There are two important parameters, *c* and γ, that require optimization: *c* represents the cost factor controlling the trade-off for maximizing the margin and minimizing the error rate, and γ regulates model generalization. In order to optimize these parameters during model training, the grid-search strategy and the GFO^98^ algorithm were used, as well as five-fold cross-validation on the training dataset, to fully optimize the model performance. In addition, we adopted the following strategies to avoid potential overfitting problems:

(i) At each cross-validation step, addition of each of the features to train the model was achieved by using four folds of the dataset, validating the performance of the trained model on the singled-out fold of the dataset. In our effort to effectively minimise the potential overfitting due to biased selection of features, the training and test datasets were kept distinct (i.e. completely separated) for each round of model validation.
(ii) During the SVR model training process, we used the model training strategies (grid search and cross-validation) recommended by the LibSVM package to optimize the relevant parameters (namely, *c* and γ), which allowed us to effectively prevent the overfitting risk by rationally separating the data samples.

### Identifying common knowledge from the source domain using the mRMR algorithm

Common knowledge (features) shared by the MMPs in the source domain was identified by feature selection using the mRMR algorithm^82^. We used mRMR to rank and select the top 50 features for all the MMPs in the source domain (5 proteases). The corresponding features were extracted from both source domain samples and target domain samples. The data was treated as the basic information for the model construction.

### Target-domain modeling based on transferred common knowledge

The initial feature set for MMP-2, -3, -7, -8, -9, and -12 substrate-cleavage site prediction (target domain) was composed of all common features (knowledge) identified from the source domain. This feature set was used to perform feature selection for each MMP (i.e., MMP-2, -3, -7, -8, -9, and -12) in the target domain. Candidate features of the MMPs in the target domain were identified by first using the mRMR algorithm to generate and rank the top 100 features for MMP-2, -3, -7, -8, -9, and -12 in the target domain. We then combined the common knowledge with each of the top-100 features listed from the target domain to generate a feature list without overlaps. The common features were located at the beginning of each feature list. A feature-selection calculation based on the six feature lists was then performed to determine the optimal feature subsets. During each step of this process, one feature was added and a SVR model was constructed before using the AUC value to evaluate model performance. Subsequent model building incorporated five-fold cross-validation and performance evaluation. After all models were completed, we chose the models for each protease of the target domain exhibiting the highest AUC value and compared the performance of the transfer-learning method with that of the feature-selection method by analyzing ROC curves and AUC values associated with five-fold cross-validation and use of the independent test dataset.

The pseudo code describing this process is shown in **Algorithm 1** below, which is composed of two sections, including common knowledge from the source domain and model training in the target domain.

#### Algorithm 1

: Framework of knowledge transfer-based model training. 

### Performance evaluation

To evaluate the predictive performance of transfer learning model versus the baseline model, six performance measures were used, including sensitivity, specificity, accuracy, F-score, MCC, and AUC. These measures are defined as follows:

2 $$Sensitivity=\,\frac{TP}{TP+FN}$$

3 $$Specificity=\,\frac{TN}{TN+FP}$$

4 $$Accuracy=\,\frac{TP+TN}{TP+TN+FP+FN}$$

5 $$F-Score=\,\frac{2\times TP}{2\times TP+FP+TN}$$

6 $$MCC=\,\frac{TP\,\times \,TN-FP\,\times \,FN}{\sqrt{(TP+FP)(TP+FN)(TN+FP)(TN+FN)}}$$

where *TP*, *TN*, *FP*, and *FN* denote the numbers of true positives, true negatives, false positives, and false negatives, respectively.

## References

[1] Antalis TM, Shea-Donohue T, Vogel SN, Sears C, Fasano A (2007). Mechanisms of disease: protease functions in intestinal mucosal pathobiology. Nat Clin Pr. Gastroenterol Hepatol.

[2] Turk B (2006). Targeting proteases: successes, failures and future prospects. Nat Rev Drug Discov.

[3] Chang HY, Yang X (2000). Proteases for cell suicide: functions and regulation of caspases. Microbiol Mol Biol Rev.

[4] Eychner AM, Lebo RJ, Elkins KM (2015). Comparison of proteases in DNA extraction via quantitative polymerase chain reaction. Anal Biochem.

[5] Overall CM, Blobel CP (2007). In search of partners: linking extracellular proteases to substrates. Nat. Rev. Mol. Cell Biol..

[6] Lopez-Otin C, Matrisian LM (2007). Emerging roles of proteases in tumour suppression. Nat Rev Cancer.

[7] Li J (2010). New insights into the evolution of subtilisin-like serine protease genes in Pezizomycotina. BMC Evol Biol.

[8] Hedstrom L (2002). Serine protease mechanism and specificity. Chem Rev.

[9] Di Cera E (2009). Serine proteases. IUBMB Life.

[10] Pham CT (2006). Neutrophil serine proteases: specific regulators of inflammation. Nat Rev Immunol.

[11] Safavi F, Rostami A (2012). Role of serine proteases in inflammation: Bowman-Birk protease inhibitor (BBI) as a potential therapy for autoimmune diseases. Exp Mol Pathol.

[12] Sharony R (2010). Protein targets of inflammatory serine proteases and cardiovascular disease. J Inflamm.

[13] Wiedow O, Meyer-Hoffert U (2005). Neutrophil serine proteases: potential key regulators of cell signalling during inflammation. J Intern Med.

[14] Pejler G, Ronnberg E, Waern I, Wernersson S (2010). Mast cell proteases: multifaceted regulators of inflammatory disease. Blood.

[15] Koblinski JE, Ahram M, Sloane BF (2000). Unraveling the role of proteases in cancer. Clin Chim Acta.

[16] Sevenich L, Joyce JA (2014). Pericellular proteolysis in cancer. Genes Dev.

[17] Maskos K, Bode W (2003). Structural basis of matrix metalloproteinases and tissue inhibitors of metalloproteinases. Mol Biotechnol.

[18] Tallant C, Marrero A, Gomis-Ruth FX (2010). Matrix metalloproteinases: fold and function of their catalytic domains. Biochim Biophys Acta.

[19] Nagase H, Visse R, Murphy G (2006). Structure and function of matrix metalloproteinases and TIMPs. Cardiovasc Res.

[20] Eckhard, U. *et al*. Active site specificity profiling of the matrix metalloproteinase family: Proteomic identification of 4300 cleavage sites by nine MMPs explored with structural and synthetic peptide cleavage analyses. *Matrix Biol*. doi:10.1016/j.matbio.2015.09.003 (2015). 10.1016/j.matbio.2015.09.003 26407638

[21] Visse R, Nagase H (2003). Matrix metalloproteinases and tissue inhibitors of metalloproteinases: structure, function, and biochemistry. Circ Res.

[22] Egeblad M, Werb Z (2002). New functions for the matrix metalloproteinases in cancer progression. Nat Rev Cancer.

[23] Nabeshima K, Inoue T, Shimao Y, Sameshima T (2002). Matrix metalloproteinases in tumor invasion: role for cell migration. Pathol Int.

[24] Palmisano R, Itoh Y (2010). Analysis of MMP-dependent cell migration and invasion. Methods Mol Biol.

[25] Newby AC (2006). Matrix metalloproteinases regulate migration, proliferation, and death of vascular smooth muscle cells by degrading matrix and non-matrix substrates. Cardiovasc Res.

[26] Page-McCaw A, Ewald AJ, Werb Z (2007). Matrix metalloproteinases and the regulation of tissue remodelling. Nat. Rev. Mol. Cell Biol..

[27] Parks WC, Wilson CL, Lopez-Boado YS (2004). Matrix metalloproteinases as modulators of inflammation and innate immunity. Nat Rev Immunol.

[28] Dandona P (2014). A mixed anti-inflammatory and pro-inflammatory response associated with a high dose of corticosteroids. Curr Mol Med.

[29] Gomez-Pina V (2012). Role of MMPs in orchestrating inflammatory response in human monocytes via a TREM-1-PI3K-NF-kappaB pathway. J Leukoc Biol.

[30] Roy S (2006). Regulation of vascular responses to inflammation: inducible matrix metalloproteinase-3 expression in human microvascular endothelial cells is sensitive to antiinflammatory Boswellia. Antioxid Redox Signal.

[31] Hao L, Du M, Lopez-Campistrous A, Fernandez-Patron C (2004). Agonist-induced activation of matrix metalloproteinase-7 promotes vasoconstriction through the epidermal growth factor-receptor pathway. Circ Res.

[32] Isenberg JS, Shiva S (2009). Vasoconstriction: tightening the noose through MMPs. Cardiovasc Res.

[33] Nugent WH, Mishra N, Strauss JF, Walsh SW (2016). Matrix Metalloproteinase 1 Causes Vasoconstriction and Enhances Vessel Reactivity to Angiotensin II via Protease-Activated Receptor 1. Reprod Sci.

[34] Lekontseva O, Jiang Y, Davidge ST (2009). Estrogen replacement increases matrix metalloproteinase contribution to vasoconstriction in a rat model of menopause. J Hypertens.

[35] Agewall S (2006). Matrix metalloproteinases and cardiovascular disease. Eur Hear. J.

[36] Lenz O, Elliot SJ, Stetler-Stevenson WG (2000). Matrix metalloproteinases in renal development and disease. J Am Soc Nephrol.

[37] Hadler-Olsen E, Fadnes B, Sylte I, Uhlin-Hansen L, Winberg JO (2011). Regulation of matrix metalloproteinase activity in health and disease. FEBS J.

[38] Malemud CJ (2006). Matrix metalloproteinases (MMPs) in health and disease: an overview. Front Biosci.

[39] Overall CM, López-Otín C (2002). Strategies for MMP inhibition in cancer: innovations for the post-trial era. Nat. Rev. Cancer.

[40] Gasparini G, Longo R, Toi M, Ferrara N (2005). Angiogenic inhibitors: a new therapeutic strategy in oncology. Nat. Clin. Pract. Oncol..

[41] Butler GS, Dean RA, Morrison CJ, Overall CM (2010). Identification of cellular MMP substrates using quantitative proteomics: isotope-coded affinity tags (ICAT) and isobaric tags for relative and absolute quantification (iTRAQ). Methods Mol Biol.

[42] Starr AE, Bellac CL, Dufour A, Goebeler V, Overall CM (2012). Biochemical characterization and N-terminomics analysis of leukolysin, the membrane-type 6 matrix metalloprotease (MMP25): chemokine and vimentin cleavages enhance cell migration and macrophage phagocytic activities. J Biol Chem.

[43] Schlage P, auf dem Keller U (2015). Proteomic approaches to uncover MMP function. Matrix Biol.

[44] Stegemann C (2013). Proteomic identification of matrix metalloproteinase substrates in the human vasculature. Circ Cardiovasc Genet.

[45] Lopez-Otin C, Overall CM (2002). Protease degradomics: a new challenge for proteomics. Nat Rev Mol Cell Biol.

[46] Prudova A, auf dem Keller U, Butler GS, Overall CM (2010). Multiplex N-terminome analysis of MMP-2 and MMP-9 substrate degradomes by iTRAQ-TAILS quantitative proteomics. Mol. Cell. Proteomics.

[47] Schilling O, Overall CM (2008). Proteome-derived, database-searchable peptide libraries for identifying protease cleavage sites. Nat. Biotechnol..

[48] Kukreja M (2015). High-Throughput Multiplexed Peptide-Centric Profiling Illustrates Both Substrate Cleavage Redundancy and Specificity in the MMP Family. Chem. Biol..

[49] Ratnikov BI (2014). Basis for substrate recognition and distinction by matrix metalloproteinases. Proc. Natl. Acad. Sci. USA.

[50] Song J (2012). PROSPER: An Integrated Feature-Based Tool for Predicting Protease Substrate Cleavage Sites. PLoS One.

[51] Song, J. *et al*. Cascleave: towards more accurate prediction of caspase substrate cleavage sites. **26**, 752–760 (2010). 10.1093/bioinformatics/btq043 20130033

[52] Wang M (2014). Cascleave 2.0, a new approach for predicting caspase and granzyme cleavage targets. Bioinformatics.

[53] Boyd, S. E., G de la Banda, M., Pike, R. N., Whisstock, J. C. & Rudy, G. B. PoPS: a computational tool for modeling and predicting protease specificity. *Proc. IEEE Comput. Syst. Bioinform. Conf*. 372–381, doi:10.1109/CSB.2004.1332450 (2004). 10.1109/csb.2004.1332450 16448030

[54] Verspurten J, Gevaert K, Declercq W, Vandenabeele P (2009). SitePredicting the cleavage of proteinase substrates. Trends Biochem. Sci..

[55] Piippo M, Lietzen N, Nevalainen OS, Salmi J, Nyman TA (2010). Pripper: prediction of caspase cleavage sites from whole proteomes. BMC Bioinformatics.

[56] Garay-Malpartida HM, Occhiucci JM, Alves J, Belizario JE (2005). CaSPredictor: a new computer-based tool for caspase substrate prediction. Bioinformatics.

[57] Backes C, Kuentzer J, Lenhof HP, Comtesse N, Meese E (2005). GraBCas: a bioinformatics tool for score-based prediction of Caspase- and Granzyme B-cleavage sites in protein sequences. Nucleic Acids Res.

[58] Wee LJ, Tan TW, Ranganathan S (2007). CASVM: web server for SVM-based prediction of caspase substrates cleavage sites. Bioinformatics.

[59] Pan SJ, Yang QA (2010). A Survey on Transfer Learning. IEEE Trans. Knowl. Data Eng..

[60] Kumar, S., Ratnikov, B. I., Kazanov, M. D., Smith, J. W. & Cieplak, P. C. CleavPredict: A platform for reasoning about matrix metalloproteinases proteolytic events. *PLoS One* **10** (2015). 10.1371/journal.pone.0127877 PMC4440711 25996941

[61] Schweikert G, Widmer C, Schölkopf B, Rätsch G (2009). An Empirical Analysis of Domain Adaptation Algorithms for Genomic Sequence Analysis. Baseline.

[62] Widmer C, Toussaint NC, Altun Y, Kohlbacher O, Rätsch G (2010). Novel machine learning methods for MHC class I binding prediction. Lecture Notes in Computer Science (including subseries Lecture Notes in Artificial Intelligence and Lecture Notes in Bioinformatics).

[63] Widmer C, Toussaint NC, Altun Y, Rätsch G (2010). Inferring latent task structure for Multitask Learning by Multiple Kernel Learning. BMC Bioinformatics.

[64] Xu Q, Pan SJ, Xue HH, Yang Q (2011). Multitask learning for protein subcellular location prediction. IEEE/ACM Trans. Comput. Biol. Bioinforma..

[65] Liu Q (2010). Multi-task learning for cross-platform siRNA efficacy prediction: an in-silico study. BMC Bioinformatics.

[66] Mei S (2012). Multi-kernel transfer learning based on Chou’s PseAAC formulation for protein submitochondria localization. J. Theor. Biol..

[67] Puniyani, K., Kim, S. & Xing, E. P. Multi-population GWA mapping via multi-task regularized regression. *Bioinformatics* **26** (2010). 10.1093/bioinformatics/btq191 PMC2881376 20529908

[68] Tamada Y (2005). Utilizing evolutionary information and gene expression data for estimating gene networks with bayesian network models. J. Bioinform. Comput. Biol.

[69] Nassar, M., Abdallah, R., Zeineddine, H. A., Yaacoub, E. & Dawy, Z. A new multitask learning method for multiorganism gene network estimation. In *IEEE International Symposium on Information Theory – Proceedings* 2287–2291, doi:10.1109/ISIT.2008.4595398 (2008).

[70] Qi Y, Tastan O, Carbonell JG, Klein-Seetharaman J, Weston J (2011). Semi-supervised multi-task learning for predicting interactions between HIV-1 and human proteins. Bioinformatics.

[71] Xu, Q., Xiang, E. W. & Yang, Q. Protein-protein interaction prediction via collective matrix factorization. in *Proceedings − 2010 IEEE International Conference on Bioinformatics and Biomedicine, BIBM 2010*. 62–67, doi:10.1109/BIBM.2010.5706537 (2010).

[72] Dahlmeier D, Ng HT (2010). Domain adaptation for semantic role labeling in the biomedical domain. Bioinformatics.

[73] Bi, J. *et al*. An Improved Multi-task Learning Approach with Applications in Medical Diagnosis. *Proc. 2008 Eur. Conf. Mach. Learn. Knowl. Discov. Databases-Part I* 117–132 (2008).

[74] Van Kasteren, T. L. M., Englebienne, G. & Kröse, B. J. A. Recognizing activities in multiple contexts using transfer learning. In *AAAI Fall Symposium - Technical Report* **FS-08-02**, 142–149 (2008).

[75] Xu Q, Yang Q (2011). A Survey of Transfer and Multitask Learning in Bioinformatics. J. Comput. Sci. Eng..

[76] Chen Z, Zhou Y, Zhang Z, Song J (2014). Towards more accurate prediction of ubiquitination sites: A comprehensive review of current methods, tools and features. Brief. Bioinform..

[77] Saeys Y, Inza I, Larrañaga P (2007). A review of feature selection techniques in bioinformatics. Bioinformatics.

[78] O’Shea JP (2013). pLogo: a probabilistic approach to visualizing sequence motifs. Nat Meth.

[79] Verma RP, Hansch C (2007). Matrix metalloproteinases (MMPs): Chemical-biological functions and (Q)SARs. Bioorganic Med. Chem..

[80] Fu L, Niu B, Zhu Z, Wu S, Li W (2012). CD-HIT: Accelerated for clustering the next-generation sequencing data. Bioinformatics.

[81] Chen Z, Zhou Y, Song J, Zhang Z (2013). hCKSAAP_UbSite: improved prediction of human ubiquitination sites by exploiting amino acid pattern and properties. Biochim. Biophys. Acta.

[82] Peng H, Ding C, Long F (2005). Minimum redundancy-maximum relevance feature selection. IEEE Intelligent Systems.

[83] Li Y (2014). Accurate in silico identification of species-specific acetylation sites by integrating protein sequence-derived and functional features. Sci. Rep..

[84] Fuchs JE (2013). Cleavage Entropy as Quantitative Measure of Protease Specificity. PLoS Comput. Biol..

[85] Rawlings ND, Barrett AJ, Finn R (2015). Twenty years of the *MEROPS* database of proteolytic enzymes, their substrates and inhibitors. Nucleic Acids Res..

[86] Kukreja M (2015). Profiling Illustrates Both Substrate Cleavage Redundancy and Specificity in the MMP Family Resource Profiling Illustrates Both Substrate Cleavage Redundancy and Specificity in the MMP Family. Chem. Biol..

[87] Trost B, Kusalik A (2013). Computational phosphorylation site prediction in plants using random forests and organism-specific instance weights. Bioinformatics.

[88] Petersen B, Petersen T, Andersen P, Nielsen M, Lundegaard C (2009). A generic method for assignment of reliability scores applied to solvent accessibility predictions. BMC Struct. Biol..

[89] Kawashima, S. *et al*. AAindex: Amino acid index database, progress report 2008. *Nucleic Acids Res*. **36** (2008). 10.1093/nar/gkm998 PMC2238890 17998252

[90] Mizianty MJ, Kurgan L (2011). Sequence-based prediction of protein crystallization, purification and production propensity. Bioinformatics.

[91] Uversky V, Gillespie J, Fink A (2000). Why are ‘natively unfolded’ proteins unstructured under physiologic conditions?. Proteins.

[92] Radivojac P (2011). Identification, Analysis and Prediction of Protein Ubiquitination Sites. Proteins.

[93] Chen Z (2011). Prediction of ubiquitination sites by using the composition of k-spaced amino acid pairs. PLoS One.

[94] Jones DT, Cozzetto D (2015). DISOPRED3: Precise disordered region predictions with annotated protein-binding activity. Bioinformatics.

[95] Bhagwat M, Aravind L (2007). PSI-BLAST tutorial. Methods Mol Biol.

[96] Peng HC, Long FH, Ding C (2005). Feature selection based on mutual information: Criteria of max-dependency, max-relevance, and min-redundancy. IEEE Trans. Pattern Anal. Mach. Intell..

[97] Chang C, Lin C (2011). LIBSVM: A Library for Support Vector Machines. ACM Trans. Intell. Syst. Technol..

[98] Lei J-B, Yin J-B, Shen H-B (2013). GFO: A data driven approach for optimizing the Gaussian function based similarity metric in computational biology. Neurocomputing.

